# The faster, the better? Relationships between run-up speed, the degree of difficulty (D-score), height and length of flight on vault in artistic gymnastics

**DOI:** 10.1371/journal.pone.0213310

**Published:** 2019-03-07

**Authors:** Christoph Schärer, Thomas Lehmann, Falk Naundorf, Wolfgang Taube, Klaus Hübner

**Affiliations:** 1 Swiss Federal Institute of Sport Magglingen (SFISM), Department of Elite Sports, Magglingen, Switzerland; 2 University of Fribourg, Department of Medicine, Movement and Sport Science, Fribourg, Switzerland; 3 Institute of Applied Training Science Leipzig (IAT), Department Strength and Technique, Leipzig, Germany; University of North Carolina at Chapel Hill, UNITED STATES

## Abstract

On vault in artistic gymnastics, a high run-up speed is thought to be important when performing difficult vaults. To test this assumption in a large cohort of elite athletes, we calculated the correlations between the run-up speed, scores, height and length of flight for handspring-, Tsukahara- and Yurchenko-style vaults and compared the performances of male and female elite and junior athletes (n = 407) during the 2016 European Championships. In females, run-up speed correlated significantly with the difficulty (D-) score and height of flight for all vaulting styles (r ≤ 0.80). In males, run-up speed correlated significantly with the D-score, height and length of flight of Tsukahara (r ≤ 0.69) and Yurchenko vaults only (r ≤ 0.65). Males reached 8–9% higher run-up speeds performing handspring and Tsukahara vaults than did females, but similar run-up speeds performing Yurchenko vaults. Elite females achieved higher run-up speeds than junior females performing Yurchenko vaults. Elite males displayed higher run-up speeds than junior males performing handspring and Tsukahara vaults. We conclude that, in females, more difficult vaults require higher run-up speeds than vaults with lower D-scores and thus, within the measured range of speeds, the faster the run-up, the better, regardless of vaulting style. Males, on the other hand, may not need to exhaust their sprinting capacity, even for the most difficult vaults. Finally, the knowledge of the required run-up speed for each vault helps coaches to estimate each athlete’s potential and/or to focus the training on developing the required physical qualities.

## Introduction

In men’s and women’s artistic gymnastics, the gymnast with the highest final score (F-score) gets to stand on top of the podium. Since the F-score is the sum of the difficulty (D-score) and the execution score (E-score), the chance of a good ranking in competition increases when a difficult routine is attempted due to the higher start value. The vault is one of six apparatus for men and one of four for women. Despite its short duration (< 5s) [1], vaulting performance can be divided into seven phases: run-up, hurdle or round off, take-off, preflight, support, post flight and landing [2]. In competition, gymnasts can freely select a vault conforming to their skill level. According to official competition rules, difficult vaults are assigned a high difficulty score (D-score) [3, 4]. The D-score is mainly influenced by the degrees of rotation around the transversal and longitudinal axes in the second flight phase [2]. Further, because the attainable amount of rotation depends on the moments of inertia [5–7], flight time and initial angular momentum, a high kinetic energy leading up to the take-off and push-off from vaulting board and table is essential for high-difficulty vaults.

**Fig 1:**
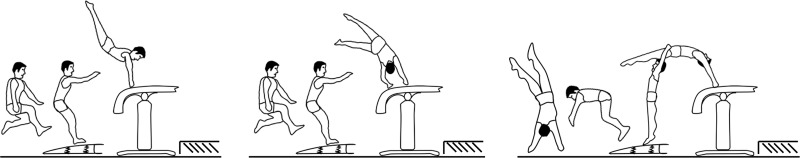
Different vault styles. The different first flight phases of the three most common vault styles in male and female artistic gymnastics. Left: Handspring; middle: Tsukahara; right: Yurchenko.

During take-off from the springboard and vaulting table, the horizontal kinetic energy that was gathered during the run-up, is converted into angular and vertical kinetic energy to facilitate an optimal second flight phase [8, 9]. Although the three most common vault styles handspring, Tsukahara (handspring with ¼ or ½ turn in the first flight phase) and Yurchenko (Round-off entry vaults (with ½ turn) in the first flight phase) (Fig 1) have different technical and physical requirements [10], it is generally acknowledged that a high run-up speed is important for a successful execution of a difficult vault. In the last decade, the vault run-up speed seems to have increased significantly [10] in men’s and women’s elite artistic gymnastics. The introduction of the new vaulting table for safety reasons in 2001, and the implementation of the unlimited scoring system in 2006 may have potentiated this development. The unlimited scoring system rewards the demonstration of difficult vaults during competitions [10]. Further, the new vaulting table increases safety by providing a much larger surface for hand placement [11] while facilitating a more anatomically functional position of the arms during the table support phase [12], thus allowing a more effective transfer of horizontal kinetic energy from the run-up phase into vertical and angular kinetic energy for the second flight phase [12].

Although the relationship between run-up speed, biomechanical parameters and the performance (D-, E- and F-score) seems logical and has been assumed for the past four decades [13], the correlations between these factors have never been calculated with a large cohort of either elite or junior gymnasts. Further, the performances of male and female, junior and elite gymnasts at a high level international competition have never been compared in terms of run-up speed, height and length of flight.

Hence, the main purpose of our study was to quantify the relationships between run-up speed and the scores (D-, E-and F-score), the height and the length of flight of the second flight phase in a high-level competition. The additional aims were to compare males and females, elite and junior gymnasts and the different vault styles handspring, Tsukahara and Yurchenko with regard to the run-up speed, the competition results (D-, E- and F-score) and the height and length of flight.

## Materials and methods

The data collection occurred during the official competitions of the 2016 European Championships in Artistic Gymnastics (EC). The measurements were performed with approval of the men’s and women’s technical committee of the European Union of Gymnastics. The data were anonymized and made available to the national gymnastics federations. The institutional review board of the Swiss Federal Institute of Sport Magglingen (SFISM) approved this study, which was conducted in compliance to the current version of the Declaration of Helsinki, the ICH-GCP or ISO EN 14155 and with all national legal and regulatory requirements.

### Participants

In total, 195 female [elite (F): n = 89; Age: 19.46, SD = 3.44 y; juniors (FJ): n = 106; Age: 14.52, SD = 0.59 y] and 212 male gymnasts [elite (M): n = 89; Age: 23.06, SD = 3.68 y; juniors (MJ): n = 123; Age: 16.86, SD = 1.17 y] performed 515 vaults during the official qualification competition of the EC. In order to qualify for the event finals, 31.4% of female and 22.6% of male participants, respectively, performed a second (different) vault.

### Procedures

**Fig 2:**
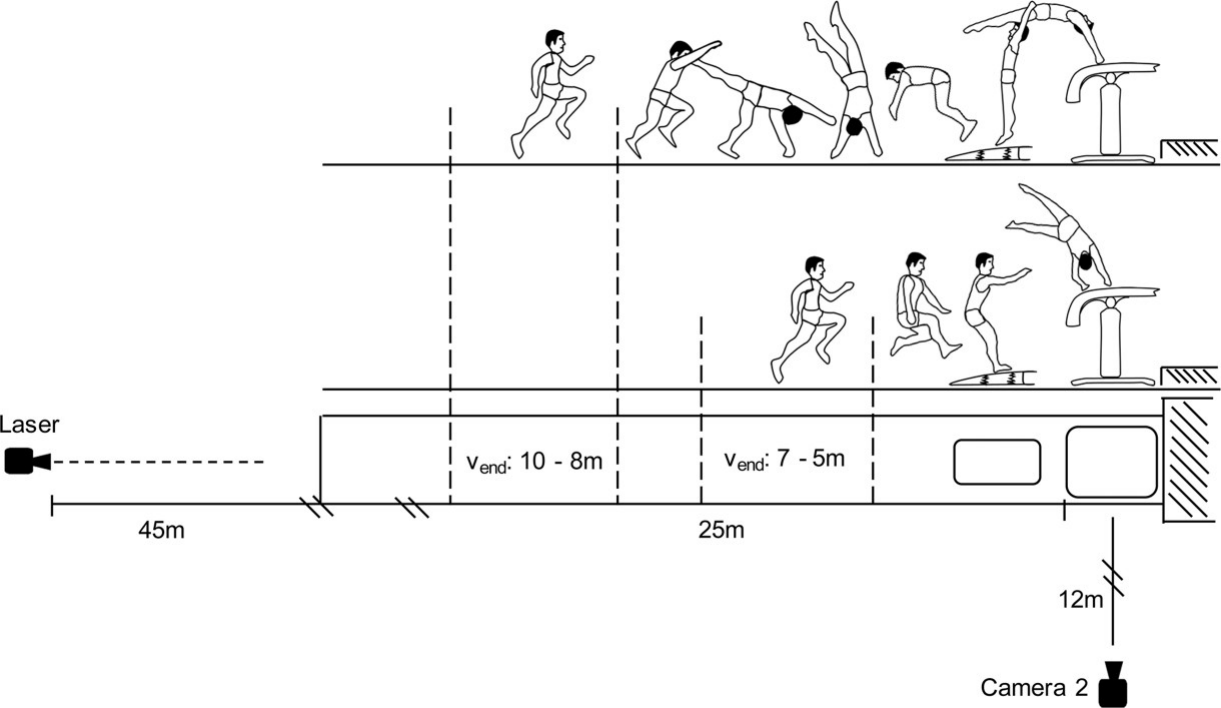
Recording conditions. Schematic representation of the recording conditions and the different measurement positions of run-up speed (v_end_) at the last step before the jump onto the springboard for Yurchenko (mean speed from 10 – 8m in front of the vaulting table) and handspring or Tsukahara vaults (mean speed from 7 – 5m) during the competitions of the 2016 European Championships in Men’s and Women’s Artistic Gymnastics.

Vault run-up speed was measured with a laser measurement system (LDM 301, Jenoptik, Rostock, Germany), placed behind the runway, 45 m from the vault table. The laser beam was aimed at the table with a height corresponding to gymnasts’ lower back. The raw (100-Hz) position data were clustered into and averaged within consecutive 0.04-s bins, thus yielding a 25-Hz position-time signal. The final run-up speed before board contact (v_end_) was then calculated at the last step before the jump onto the springboard for handspring and Tsukahara (mean speed from 7 to 5 m in front of the vaulting table) and the last running step before the round-off for Yurchenko (mean speed from 10 to 8 m in front of the vaulting table), respectively [10] (Fig 2). D- (Difficulty), E- (Execution) and F-score (Final) were taken from the official ranking list [14, 15].

Maximal height (h_max_) and length (L) of flight were detected with two synchronized full HD cameras (Basler acA 1920–155 uc, USB 3.0, Basler AG, Ahrensburg, Germany; 100 fps; 1920x1200), placed orthogonal to each other in the spectator area. Camera one was behind the vault runway (distance: 45 m); camera two was beside the vaulting table (distance: 12 m and 5 m above). The motion volume was calibrated in front of and behind the vaulting table by digitizing a 3-dimensional calibration cube (1.89 x 1.89 x 2.26 m). Video analysis was run with Simi Motion 2D/3D (Simi Reality Motion System, Unterschleissheim, Germany). Based on the coordinates of the center of mass, h_max_ was determined, whereas L was determined based on the position of the ankle, both relative to the defined point of origin. The ankle was labeled at the moment of landing and L was measured from the point intersecting the surface of the landing mat and the plumb line at the edge of the vaulting table. Interrater-reliability of tagging h_max_ and L was assessed according to Hopkins (2000) [16] with randomly chosen vaults (n = 25) and revealed a high accuracy of determining h_max_ (Pearson’s correlation coefficient: r = 0.92) and L (r > 0.99), a small typical error (TE) (h_max_: 0.07 m; L: 0.02 m), no systematic error (t-test) (h_max_: p = 0.90; L: p = 0.17) and small random errors (relative coefficient of variation: CV %) (h_max_: CV% = 3.33%; L: CV% = 0.85%).

### Statistical analyses

Descriptive statistics were run on all variables separately for the four groups (elite and junior, male and female) as well as for the vaulting styles handspring, Tsukahara and Yurchenko. Boxplots of v_end_ were constructed for each group to define the range of the optimal run-up speed (between lower to upper quartiles) for the different vaults (n ≥ 3). The relationships between run-up speed, D-, E- and F-Score, height and length of flight were assessed using Spearman’s Rho and explained variance (R^2^). Mann-Whitney-U-Tests were used to determine differences between genders, age groups and vaulting styles. Effect sizes (ES) were calculated by dividing the standardized test statistic (z) by the square root of N (total number of cases) and classified as either small (< 0.1), medium (> 0.3) or large (> 0.5) using the criteria according to Cohen [17]. Significance level was set to p < 0.05. All statistics were performed using SPSS 22 software (SPSS Inc., Chicago, IL).

## Results

In 21 of the 515 vaults, either v_end_, h_max_ or L could not be determined due to disturbances by spectators and/or recording problems. The remaining 494 vaults were included in the data analysis.

### Male vs. female athletes

Altogether, 51% of vaults presented by females were Yurchenko-style vaults, 24.5% were handspring and 24.5% were Tsukahara vaults. Males predominantly performed Tsukahara (69%), followed by handspring (19%) and Yurchenko vaults (12%).

When performing handspring and Tsukahara vaults, males displayed significantly higher run-up speeds (elite: + 9%; juniors: + 8%; p < 0.001; ES > 0.61) as well as greater h_max_ (elite: + 17%; juniors: + 12%; p < 0.001; ES > 0.66) and L (elite: +37%; juniors: +18%; p < 0.001; ES > 0.49) than females in their respective age category. In contrast, the run-up speeds for Yurchenko did not differ between males and females (elite: p = 0.32; juniors: p = 0.18; ES < 0.21). Nonetheless, h_max_ (+ 9%) and L (+ 16%) were significantly greater in male elite than female elite gymnasts (p < 0.01; ES > 0.36). Gymnasts performing two vaults displayed significantly lower run-up speed when performing Yurchenko compared to handspring or Tsukahara, regardless of gender or age category (all p < 0.05). However, the decrease in run-up speed was more severe for males (elite: -10.52%; juniors: -8.48%) than for females (elite: - 3.60%; juniors: -5.92%).

### Elite vs. junior athletes

Mean values and statistical differences between elite and junior females and between elite and junior males for all measured parameters are displayed in Tables 1 and 2.

**Table 1:** Descriptive statistics females.

Category	n	v_end_ ± SD	h_max_ ± SD	L ± SD	D-score ± SD	E-score ± SD	F-score ± SD
Handspring F	28	7.63 ± 0.32	2.41 ± 0.15	1.84 ± 0.30	4.89 ± 0.46**	8.62 ± 0.55	13.48 ± 0.95*
Handspring FJ	32	7.54 ± 0.31	2.41 ± 0.11	1.95 ± 0.33	4.59 ± 0.24	8.62 ± 0.40	13.17 ± 0.54
Tsukahara F	25	7.40 ± 0.38	2.28 ± 0.13	1.81 ± 0.28	4.50 ± 0.42*	8.41 ± 0.47	12.90 ± 0.78
Tsukahara FJ	35	7.23 ± 0.33	2.29 ± 0.07	1.79 ± 0.29	4.29 ± 0.26	8.47 ± 0.27	12.76 ± 0.44
Yurchenko F	62	7.31 ± 0.25*	2.49 ± 0.15	1.83 ± 0.2	5.10 ± 0.50*	8.85 ± 0.34*	13.93 ± 0.73**
Yurchenko FJ	64	7.19 ± 0.21	2.44 ± 0.09	1.81 ± 0.31	4.69 ± 0.95	8.46 ± 1.54	13.13 ± 2.43

Mean values, standard deviation and statistical difference (Mann-Whitney-U-Test) between female elite and junior athletes of the run-up speed, height and length of flight and D-, E- and F-score on vault (n = number of participants; v_end_ = run-up velocity at the last step before the jump onto the springboard; h_max_ = maximal height of flight; L = length of flight; D-score = Difficulty score; E-score = Execution score; F-score = Final score; F = Female elite athletes; FJ = Female junior athletes

*: F is different from FJ (p < 0.05)

**: F is different from FJ (p < 0.01)).

**Table 2:** Descriptive statistics males.

Category	n	v_end_ ± SD	h_max_ ± SD	L ± SD	D-score ± SD	E-score ± SD	F-score ± SD
Handspring M	23	8.45 ± 0.28**	2.87 ± 0.16**	2.53 ± 0.32	5.57 ± 0.38**	8.98 ± 0.38*	14.51 ± 0.58***
Handspring MJ	25	8.20 ± 0.33	2.74 ± 0.11	2.51 ± 0.46	4.74 ± 0.97	8.77 ± 0.49	13.50 ± 0.96
Tsukahara M	63	8.19 ± 0.32***	2.68 ± 0.14	2.56 ± 0.39***	5.26 ± 0.53***	8.86 ± 0.36	14.06 ± 0.75***
Tsukahara MJ	108	7.88 ± 0.35	2.56 ± 0.15	2.31 ± 0.46	4.58 ± 0.64	8.82 ± 0.32	13.37 ± 0.75
Yurchenko M	16	7.41 ± 0.34	2.72 ± 0.12**	2.13 ± 0.36	5.35 ± 0.44***	9.02 ± 0.23	14.33 ± 0.45***
Yurchenko MJ	13	7.31 ± 0.33	2.58 ± 0.12	1.91 ± 0.44	4.92 ± 0.38	8.83 ± 0.33	13.69 ± 0.49

Mean values, standard deviation and statistical difference (Mann-Whitney-U-Test) between male elite and junior athletes of the run-up speed, height and length of flight and D-, E- and F-score on vault (n = number of participants; v_end_ = run-up speed at the last step before the jump onto the springboard; h_max_ = maximal height of flight; L = length of flight; D-score = Difficulty score; E-score = Execution score; F-score = Final score; M = Male elite athletes; MJ = Male junior athletes

*: M is different from MJ (p < 0.05)

**: M is different from MJ (p < 0.01)

***: M is different from MJ (p < 0.001)).

**Fig 3:**
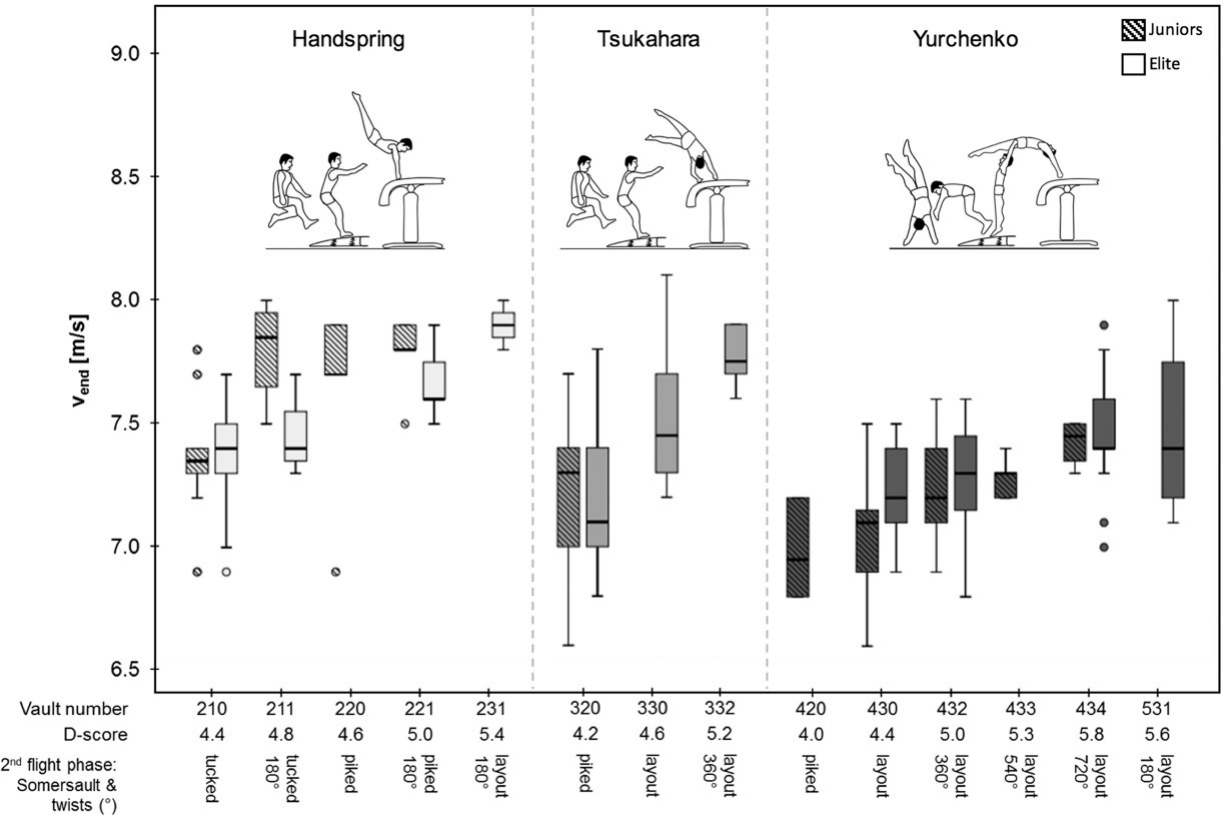
Females’ optimal run-up speed. Boxplots of the run-up speed (v_end_) of elite and junior female athletes separated into the different vault styles (handspring, Tsukahara, Yurchenko) and vaults (vault numbers) performed during the qualification of the 2016 European Championships in Men’s and Women’s Artistic Gymnastics.

**Fig 4:**
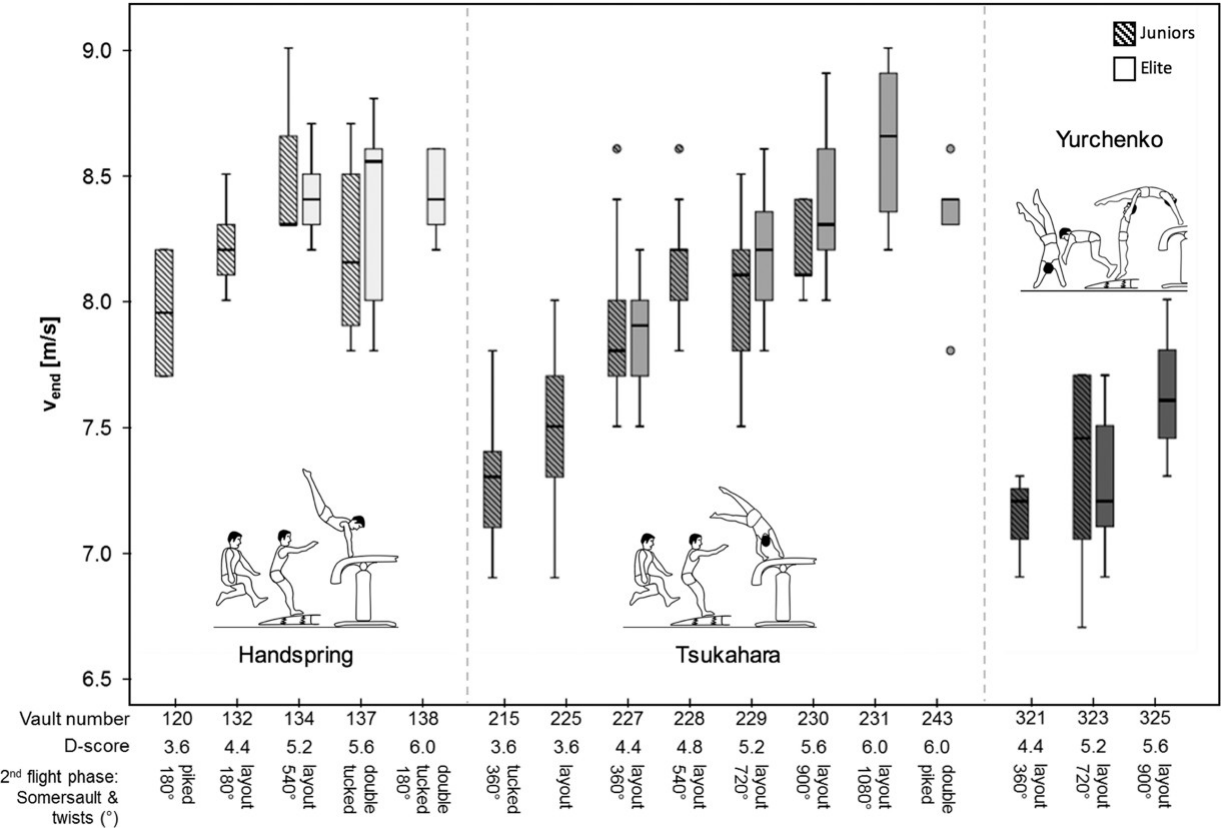
Males’ optimal run-up speed. Boxplots of the run-up speed (v_end_) of elite and junior male athletes separated into the different vault styles (handspring, Tsukahara, Yurchenko) and vaults (vault numbers) performed during the qualification of the 2016 European Championships in Men’s and Women’s Artistic Gymnastics.

Boxplots of the run-up speed (separated into elite and junior categories) show the variation of v_end_ for the different vaults (Figs 3 and 4). Elite and junior gymnasts performing identical vaults did so with a similar v_end_ (all p > 0.05).

For male and female elite and junior athletes, v_end_ correlated better with the D-score than with E- and F-Scores and better with h_max_ than with L (Table 3). Differences in v_end_ explained 64% (females) and 42% (males) of the variation in D-scores for females and males, respectively, whereas h_max_ explained 56% and 47% (males) of variation, respectively.

**Table 3:** Correlations.

v_end_	n	D-score	E-score	F-score	h_max_	L
Handspring F	28	0.80**	0.60**	0.74**	0.75*	0.00
Tsukahara F	25	0.72**	0.73**	0.79**	0.63**	0.58**
Yurchenko F	62	0.39**	0.15	0.31*	0.30*	0.22
Handspring FJ	32	0.65**	0.35*	0.57**	0.70**	0.08
Tsukahara FJ	35	0.53**	0.29	0.38*	0.58**	0.48**
Yurchenko FJ	64	0.48**	0.33**	0.46**	0.44**	0.29*
Handspring M	23	0.14	0.13	0.17	0.33	0.05
Tsukahara M	63	0.60**	0.43**	0.64**	0.69**	0.51**
Yurchenko M	16	0.65**	0.03	0.51*	0.46	0.54*
Handspring MJ	10	0.31	0.00	0.33	0.39	0.24
Tsukahara MJ	8	0.60**	0.19*	0.50**	0.57**	0.65**
Yurchenko MJ	13	0.27	0.15	0.37	0.00	0.61*

Correlation coefficients (Spearman’s Rho) between run-up speed D-, E- and F-score as well as height and length of flight of male and female elite and junior athletes on vault (D-score = Difficulty score; E-score = Execution score; F-score = Final score; v_end_ = run-up speed at the last step before the jump onto the springboard; h_max_ = maximal height of flight; L = length of flight; F = Female elite athletes; FJ = Female junior athletes; M = Male elite athletes; MJ = Male junior athletes

*: Significance at p < 0.05

**: Significance at p < 0.01).

## Discussion

This is the first study to investigate the relationships between run-up speed and competition scores as well as height and length of flight on vault with a large cohort of international world-class athletes in artistic gymnastics. The long-held assumption of a strong relationship between run-up speed and the D-score on vault was confirmed in female gymnasts but appeared to be only conditionally true for males. Furthermore, run-up speed, height and length of flight were compared for the first time among males, females, juniors and elites within a very large sample during a high-level international competition.

### Correlations

In general, vault run-up speed and D-, E- and F-scores correlated stronger in females than in males. Up to 64% (females) and 42% (males) of variation in D-score (R^2^) could be explained by v_end_. Consequently, a high run-up speed is a potentially stronger limiting factor of competition performance for female than for male gymnasts. Furthermore, since women may have less relative explosive power in the upper body [18] female gymnasts may need to generate a higher percentage of their final (in-flight) kinetic energy during the run-up. In contrast, male gymnasts seem to have a wider margin of error in adapting their run-up speed with respect to their maximum running speed and may be better able to augment kinetic energy at the jump and push-off due to superior explosive power in the upper body. For both male and female gymnasts, however, the question remains as to whether a higher run-up speed could further improve their performance or if a higher run-up speed would interfere with the push-off generated from the upper body.

In junior categories, the relationship between run-up speed and the other measured parameters tended to be lower than for the elite categories. The largest difference was observed for the correlation between run-up speed and E-scores. For mostly subjective reasons, the E-score given by the judges is influenced by the run-up speed and the height of the second flight phase [19]. Further, the E-score depends on the execution of twists and somersaults during flight and the quality of landing. Landing quality is influenced by the height of the center of mass above the landing mat at the first floor contact [20], a factor which benefits from a greater height of flight. Thus, when aiming for a successful transition from the junior to the elite level on vault, it seems crucial to increase run-up speed in order to increase flight time and height of flight and consequently improve the execution of the landing.

### Male vs. female athletes

The results show that male athletes reach 8–9% higher run-up speeds performing handspring or Tsukahara vaults than do female gymnasts of the same age group. This seems to correspond a general gender difference in sprint speed, similar to that observed in track and field sprint athletes [21, 22]. Gender differences in sprint speed are of a biological origin. In particular, higher relative maximal strength and power result in better sprint speed of male athletes. Nevertheless, it must be taken into consideration that female gymnasts are in general smaller and lighter than male gymnasts. Thus, the total amount of energy that has to be generated is smaller for females than for males when performing the same vault. In contrast, female and male athletes reach similar speeds when performing Yurchenko vaults. This may imply that Yurchenko vaults only require a certain minimum v_end_ and that this minimum seems to be the same for both genders. However, this also means that females must attain run-up speeds closer to their maximal sprinting speed (only 6% lower compared to handspring or Tsukahara; males: -10%) in order to perform these vaults. It might therefore be assumed that women have to take greater risks when executing these technically demanding vaults.

Males reach significantly greater heights and lengths of flight performing all vault styles than females, even though they display a similar run-up speed performing Yurchenko vaults. This demonstrates that creating flight height does not only depend on the run-up speed [23], but also strongly depends on the ability to push-off explosively from the vaulting table. Thus, it is reasonable to assume that the greater explosive strength in the upper body accounts at least partly for this. In addition, male athletes’ vault table is slightly higher (10 cm).

Comparing the F-scores between groups, there were only small differences between elite and junior females but much greater gaps between elite and junior males. In males, these differences are mostly due to the higher D-scores (of all vault styles) of the elite gymnasts. In contrast, elite females only achieve higher D- and E-scores than juniors when performing Yurchenko vaults.

To summarize differences between male and female athletes, male athletes have a greater ability to produce and use muscular force and are able to create more horizontal and/or vertical kinetic energy, which enables them to demonstrate vaults with greater degrees of difficulty. However, it is important to note that run-up speed accounts only partly for these differences.

### Elite vs. junior athletes

Comparing the run-up speeds of elite and junior gymnasts performing identical vaults revealed no statistical differences. Consequently, it can be concluded that the optimal run-up speed for each vault is independent of age.

In general, there were only small differences found between elite and junior females in terms of run-up speed, height and length of flight, for all vault styles. It has been previously described how growth and weight gain during puberty negatively influence the strength-to-weight ratio [24, 25]. Consequently, younger female gymnasts may have similar or even better physical prerequisites for a good performance on vault than more mature gymnasts. Only v_end_ and h_max_ of Yurchenko vaults were higher for female elite compared to female junior athletes. Therefore, we assume that not physical abilities but the technical skill level is the limiting factor of the run-up speed of these vaults [26]. Hence, a superior technique executing the round-off and back handspring probably permits elite female gymnasts to run up with a higher speed than junior females, which in turn improves the height of flight of their vaults, allowing them to perform more difficult vaults.

Contrary to the findings in females, male elite athletes had significantly higher v_end_ and h_max_ than junior gymnasts except when performing Yurchenko vaults. The reason for this is most likely related to the different and later biological development of males. Namely, there is a considerable increase in muscle mass between the junior and elite ages, which positively influences power and strength in general and sprinting abilities in particular. Aside from better technical skills [27] the improved sprinting ability and greater strength and power of the upper extremities may help explain the greater flight height in elite compared to junior male athletes. When it comes to Yurchenko vaults, it seems that males are able to attain the optimal run-up speed for Yurchenko vaults already as juniors, and that further increases in flight height and difficulty at the elite level are attained via improvements in upper body strength and technical skill.

### Vault styles

#### Handspring vaults

Of all vaulting styles, handspring vaults are generally executed with the greatest run-up speed. Consequently, only gymnasts who can realize fast run-up speeds performed handspring vaults at the EC.

Further, no significant correlations were found between the run-up speed of handspring vaults and either the height of flight, D-, E-, or F-score for males. Handspring vaults must be distinguished into double rotation and layout vaults with turns around longitudinal axis. For layout somersaults compared to double somersaults in tucked position, a higher angular momentum has to be created at take-off and push-off from the vaulting board and table due the body’s greater moment of inertia in the layout position [7]. In this context, similar run-up speeds were observed for double rotation handspring vaults and layout handspring vaults with 540° or only 180° of rotation, although the latter are assigned lower D-scores. Based on this consideration, one could suppose that training a double rotation handspring vault is more beneficial in terms of increasing the D-score than training layout vaults, since the latter require the same run-up speed but afford lower D-scores.

#### Tsukahara vaults

Tsukahara vaults are the most common vaults among males, but the least common in female gymnasts. Compared to the other vaulting styles, Tsukahara vaults showed the greatest difference in run-up speed between simple and very difficult vaults, indicating the greatest dependence on run-up speed for increasing vault difficulty. This should be taken into account by coaches and athletes when the aim is to learn a more difficult Tsukahara vault.

Across all groups and especially in female categories, the height of the second flight phase of Tsukahara vaults is lower than that of handspring or Yurchenko vaults. The asynchronous hand contact on the vaulting table during Tsukahara vaults allow less horizontal momentum to be transformed into vertical momentum and more horizontal momentum to be preserved [28], which may also explain the significant correlation between v_end_ and L in all categories. Further, the push-off from the vaulting table, because it occurs mainly with one arm, makes it difficult for athletes with less upper body strength to reach sufficient height of flight in order to perform a difficult vault. This could be the main reason that female athletes only rarely perform Tsukahara vaults.

#### Yurchenko vaults

Most females but only a few male gymnasts performed a Yurchenko vault. Compared to the other vault styles, Yurchenko vaults are performed with the lowest run-up speed. One explanation for this is certainly the truncated run-up distance due to the round-off executed in front of the springboard. Moreover, to perform the round-off and back handspring with a high velocity requires strong mental coping strategies due to the fact that the athletes do not see the vaulting table until just before the moment of push-off. Thus, anxiety might be another limiting factor for a high run-up speed. In addition, there was no difference in run-up speed between male and female gymnasts performing Yurchenko vaults. Since the upper limbs have a limiting mechanical behavior of locomotion [29], the maximum speed executing a round-off in front of the springboard may be restrained to a similar level for male and female gymnasts.

In consideration of the fact that female athletes reached higher flight heights with Yurchenko vaults compared to other vaulting styles, it can be deduced that the round-off in front of the springboard helps them to generate more angular momentum and vertical velocity than with other vault styles, despite a lower run-up speed.

#### Limitations

In order to draw definitive conclusions on the importance of a high sprint-speed potential for a fast run-up speed and to perform difficult vaults, not only the run-up speed on vault but also the maximal 25m-sprint speed of the gymnasts should be included to the evaluations in future studies. Further, we only verified the hypothesis of linear relationships between the run-up speed and the other measured parameters. Further research should aim to calculate non-linear models in particular for handspring and Yurchenko vaults in males. Moreover, future studies should pay more attention on the undisputed importance of the push-off from the vaulting table and the consecutive height of the second flight phase for the landing characteristics and for the scores. In this manner, the requirements of successful vaulting could be described even more detailed.

## Conclusions

For female athletes, it can be concluded that, within the measured range of speeds, the faster the run-up, the better (in terms of D-, E- and F-score). For males on the other hand, although a strong relationship exists between run-up speed and performance for Tsukahara vaults, a certain minimum run-up speed seems indispensable, but not necessarily more in order to perform the most difficult vaults. Nevertheless, this study confirmed that a high run-up speed is one of the most important determining factors to succeed on vault in women’s and men’s artistic gymnastics competition. Further, we show that there is an optimal range of run-up speeds for each vault. This knowledge is important for coaches and athletes when choosing a competition vault for each gymnast with regard to their physical and technical abilities. This may help to focus the training regime on developing physical qualities (strength, explosiveness, sprint technique) in order to reach the required run-up speed, if necessary. Finally, male athletes have physical advantages that permit them to perform more difficult vaults than women. At the same time, female gymnasts are especially good at using their potential energy during Yurchenko vaults due to their excellent technique and their mental capacity to cope with the backwards approach of the table despite near maximum speed.

## Supporting information

S1 Table Data table containing all measured parameters of all participants. (PDF)
