# Supplementary material for: The faster, the better? Relationships between run-up speed, the degree of difficulty (D-score), height and length of flight on vault in artistic gymnastics
Source: PLoS One. 2019 Mar 7;14(3):e0213310. doi: 10.1371/journal.pone.0213310 (PMC6405201; doi:10.1371/journal.pone.0213310)
Supplement: S1 Table — (PDF) [file pone.0213310.s001.pdf]

Athlete Nr	Gender	Category	Age	Vault Nr	Vault Group	D-Score	E-Score	Penalty	F-Score	v_{end}	h_{max}	L
Athlete 274	male	junior	16.10	114	Handspring	3.2	8.866		12.066	8	2.61	3.49
Athlete 354	male	junior	16.86	114	Handspring	3.2	8.8		12	8.2	2.65	3.35
Athlete 281	male	junior	15.36	119	Handspring	3.2	9.2		12.4	8.1	2.84	2.87
Athlete 360	male	junior	16.44	119	Handspring	3.2	9		12.2	7.5	2.51	1.70
Athlete 291	male	junior	15.47	120	Handspring	3.6	9.1		12.7	8.2	2.73	2.39
Athlete 329	male	junior	14.53	120	Handspring	3.6	9.066		12.666	7.7	2.61	1.68
Athlete 383	male	junior	18.21	120	Handspring	3.6	9		12.6		2.91	2.29
Athlete 199	male	junior	17.62	132	Handspring	4.4	8.933		13.333	8.2	2.82	2.57
Athete 214	male	elite	21.34	132	Handspring	4.4	8.9	-0.1	13.2	8.5	2.73	2.13
Athlete 257	male	junior	17.84	132	Handspring	4.4	9.066		13.466	8	2.55	2.38
Athlete 377	male	junior	18.03	132	Handspring	4.4	9.133		13.533	8.5	2.65	2.25
Athlete 385	male	junior	17.00	132	Handspring	4.4	8.458		12.858	8.1	2.58	2.43
Athlete 395	male	junior	16.94	132	Handspring	4.4	8.066	-0.1	12.366	8.3	2.66	2.35
Athete 221	male	junior	17.98	134	Handspring	5.2	8.8	-0.1	13.9	8.3	2.64	2.49
Athlete 242	male	elite	19.57	134	Handspring	5.2	9		14.2	8.2	2.75	3.19
Athlete 279	male	junior	18.17	134	Handspring	5.2	9.033		14.233	8.3	2.78	2.57
Athlete 335	male	elite	25.97	134	Handspring	5.2	8.833		14.033	8.4	2.47	2.81
Athlete 336	male	junior	16.81	134	Handspring	5.2	8.8		14	9	2.78	2.71
Athlete 344	male	elite	26.55	134	Handspring	5.2	9		14.2		2.51	2.13
Athlete 372	male	elite	19.17	134	Handspring	5.2	9.2		14.4	8.5	2.73	2.70
Athlete 388	male	elite	26.51	134	Handspring	5.2	8.8	-0.1	13.9	8.3	2.70	2.92
Athlete 402	male	elite	18.56	134	Handspring	5.2	9.133		14.333	8.7	2.91	2.64
Athlete 260	male	elite	20.53	135	Handspring	5.6	9.3	-0.1	14.8	8.6	2.78	2.28
Athete 234	male	elite	23.81	136	Handspring	6	9.3		15.3	9		
Athlete 352	male	junior	17.82	136	Handspring	6	9.4		15.4	8.4	2.79	2.28
Athete 197	male	junior	18.00	137	Handspring	5.6	7.766		13.366	8.1	2.79	2.64
Athete 200	male	elite	26.58	137	Handspring	5.6	9.333		14.933	8.5	2.83	2.26
Athete 222	male	elite	22.68	137	Handspring	5.6	8.666		14.266	8.6	2.95	2.55
Athete 229	male	elite	25.80	137	Handspring	5.6	7.9	-0.1	13.4	8.2	2.97	2.69
Athlete 245	male	junior	15.79	137	Handspring	5.6	7.633		13.233	8.1	2.85	3.24
Athlete 252	male	elite	24.57	137	Handspring	5.6	8.3	-0.1	13.8	8.8	2.96	2.66
Athlete 261	male	elite	25.47	137	Handspring	5.6	9.341		14.941	8	2.91	2.13
Athlete 262	male	junior	18.13	137	Handspring	5.6	9.033		14.633	7.9	2.75	2.34
Athlete 280	male	junior	18.23	137	Handspring	5.6	9.066		14.666	8.2	2.84	2.41
Athlete 292	male	elite	31.79	137	Handspring	5.6	9.216		14.816	8.7	3.01	2.66
Athlete 298	male	junior	18.37	137	Handspring	5.6	8.066		13.666	8.5	2.80	3.19
Athlete 312	male	elite	24.19	137	Handspring	5.6	9.1		14.7	7.8	2.90	1.92
Athlete 315	male	junior	17.96	137	Handspring	5.6	9.2		14.8	8.4	2.92	1.90
Athlete 346	male	junior	17.76	137	Handspring	5.6	7.8		13.4	8.7	2.84	2.52
Athlete 353	male	junior	18.39	137	Handspring	5.6	8.9		14.5	7.9	2.76	1.97
Athlete 358	male	elite	20.88	137	Handspring	5.6	9.366		14.966	8.6	2.90	2.60
Athlete 366	male	junior	17.24	137	Handspring	5.6	9.033		14.633	8.6	2.85	2.87
Athlete 376	male	elite	22.18	137	Handspring	5.6	8.633		14.233	8	2.79	2.44
Athlete 380	male	elite	27.29	137	Handspring	5.6	9.133		14.733	8.6	3.06	2.33
Athlete 405	male	junior	18.31	137	Handspring	5.6	8.7		14.3	7.8	2.85	2.52
Athlete 244	male	elite	35.46	138	Handspring	6	8.658	-0.1	14.558	8.2	2.88	3.20
Athlete 275	male	elite	19.36	138	Handspring	6	9	-0.1	14.9	8.6	2.95	2.41
Athlete 326	male	elite	19.29	138	Handspring	6	9.333		15.333	8.3	3.10	2.48
Athlete 349	male	elite	23.61	138	Handspring	6	9.3		15.3	8.4	3.03	2.33
Athlete 398	male	elite	22.67	138	Handspring	6	9.3		15.3	8.6	2.92	2.79
Athlete 321	male	elite	26.15	140	Handspring	6	8.333	-0.1	14.233	8.6	3.10	2.48
Athlete 17	female	Elite	17.16	210	Handspring	4.4	7.733		12.133	7.5	2.22	1.64
Athlete 58	female	Elite	17.98	210	Handspring	4.4	7.033	-0.3	11.133	7.4	2.16	2.09
Athlete 94	female	Elite	20.11	210	Handspring	4.4	8.733		13.133	7.5	2.46	2.18
Athlete 95	female	Elite	16.74	210	Handspring	4.4	8.833		13.233	7.4	2.39	1.64
Athlete 98	female	Elite	23.45	210	Handspring	4.4	8.666		13.066	7.7	2.40	2.06
Athlete 103	female	Elite	24.67	210	Handspring	4.4	7.5	-0.3	11.6	7		
Athlete 117	female	Elite	16.48	210	Handspring	4.4	7.2	-0.1	11.5	7.3	2.29	2.34
Athlete 124	female	Elite	17.29	210	Handspring	4.4	8.9		13.3	7.4	2.28	1.65
Athlete 191	female	Elite	24.14	210	Handspring	4.4	8.608		13.008	6.9	2.25	2.07
Athlete 13	female	Junior	14.19	210	Handspring	4.4	8.733	-0.3	12.833	7.7	2.52	1.93
Athlete 16	female	Junior	14.79	210	Handspring	4.4	7.766	-0.3	11.866	7.8	2.45	2.48
Athlete 29	female	Junior	14.94	210	Handspring	4.4	7.566	-0.1	11.866	7.3	2.27	1.88
Athlete 39	female	Junior	13.96	210	Handspring	4.4	8.366	-0.1	12.666	7.4	2.30	2.52
Athlete 46	female	Junior	13.74	210	Handspring	4.4	8.933		13.333	7.4	2.41	1.69
Athlete 63	female	Junior	13.98	210	Handspring	4.4	8.6		13	7.3	2.36	2.32
Athlete 68	female	Junior	15.21	210	Handspring	4.4	8.4		12.8	7.7	2.44	1.88
Athlete 83	female	Junior	14.02	210	Handspring	4.4	8.766		13.166	7.2	2.33	2.43
Athlete 90	female	Junior	14.92	210	Handspring	4.4	8.7	-0.1	13	7.3	2.35	1.99
Athlete 102	female	Junior	15.20	210	Handspring	4.4	8.9		13.3	7.8	2.67	1.85
Athlete 116	female	Junior	15.02	210	Handspring	4.4	8.666		13.066	7.3	2.32	1.85
Athlete 119	female	Junior	14.15	210	Handspring					6.9	2.22	2.17
Athlete 144	female	Junior	13.70	210	Handspring	4.4	8.8		13.2	7.4	2.37	1.95
Athlete 166	female	Junior	13.52	210	Handspring	4.4	8.5		12.9	7.3	2.35	1.39
Athlete 167	female	Junior	14.41	210	Handspring	4.4	8.933		13.333	7.4	2.49	1.50
Athlete 174	female	Junior	14.84	210	Handspring	4.4	8.7		13.1	7.3	2.30	1.44
Athlete 182	female	Junior	14.35	210	Handspring	4.4	8.8		13.2	7.4	2.37	2.27
Athlete 188	female	Junior	14.87	210	Handspring	4.4	7.766		12.166	6.9	2.44	2.18
Athlete 2	female	Elite	18.02	211	Handspring	4.8	8.8		13.6	7.4	2.35	2.03
Athlete 159	female	Elite	20.65	211	Handspring	4.8	8.633		13.433	7.3		
Athlete 176	female	Elite	16.25	211	Handspring	4.8	8.233		13.033	7.7	2.53	1.88
Athlete 187	female	Elite	15.51	211	Handspring	4.8	8.566		13.366	7.4	2.31	1.36
Athlete 57	female	Junior	14.77	211	Handspring	4.8	8.4	-0.1	13.1	7.9	2.36	2.00
Athlete 64	female	Junior	15.09	211	Handspring	4.8	8.466		13.266	7.5	2.46	1.39
Athlete 66	female	Junior	14.82	211	Handspring	4.8	8.666		13.466	7.8	2.41	2.16
Athlete 155	female	Junior	14.60	211	Handspring	4.8	9.1		13.9	8	2.45	2.39
Athlete 75	female	Elite	23.24	212	Handspring	5.3	8.933		14.233	8.2	2.60	2.13
Athlete 92	female	Elite	23.11	212	Handspring	5.3	9.083		14.383	7.9	2.61	1.92
Athlete 290	male	junior	15.82	213	Tsukahara	2.8	8.433	-0.1	11.133	7	2.26	1.06
Athete 205	male	junior	15.41	215	Tsukahara	3.6	8.966		12.566		2.62	2.42
Athlete 211	male	junior	16.01	215	Tsukahara	3.6	8.9		12.5	7.8	2.51	2.14
Athlete 253	male	junior	15.24	215	Tsukahara	3.6	8.883		12.483	7.1	2.34	1.58

Athlete Nr	Gender	Category	Age	Vault Nr	Vault Group	D-Score	E-Score	Penalty	F-Score	v_{end}	h_{max}	L
Athlete 256	male	junior	13.42	215	Tsukahara	3.6	8.45		12.05	7.3	2.41	1.44
Athlete 264	male	junior	15.74	215	Tsukahara	3.6	8.766		12.366	7.4	2.28	1.70
Athlete 384	male	junior	13.82	215	Tsukahara	3.6	8.466		12.066	6.9	2.32	1.41
Athlete 269	male	elite	18.77	216	Tsukahara	4	8.875		12.875	7.3	2.34	1.61
Athlete 370	male	elite	19.58	216	Tsukahara	4	8.591	-0.1	12.491	7.8	2.53	2.07
Athlete 283	male	junior	14.99	219	Tsukahara	3	9.1		12.1	7.7	2.65	2.52
Athlete 373	male	junior	14.55	219	Tsukahara	3	8.433	-0.3	11.133	7	2.23	1.74
Athlete 62	female	Elite	20.12	220	Handspring	4.6	8.933		13.533	7.8	2.39	2.24
Athlete 143	female	Elite	16.03	220	Handspring	4.6	9.066		13.666	7.5	2.30	2.14
Athlete 102	female	Junior	15.20	220	Handspring	4.6	8.833		13.433	7.9	2.62	2.16
Athlete 76	female	Junior	14.81	220	Handspring	4.6	8.833		13.433	7.9	2.57	2.33
Athlete 110	female	Junior	14.95	220	Handspring	4.6	8.966		13.566	7.7	2.41	2.02
Athlete 188	female	Junior	14.87	220	Handspring	4.6	8.533		13.133	6.9	2.21	1.69
Athlete 190	female	Junior	13.59	220	Handspring	4.6	9.066		13.666	7.7	2.49	1.66
Athlete 14	female	Elite	18.16	221	Handspring	5	8.633		13.633	7.6	2.45	1.27
Athlete 18	female	Elite	24.31	221	Handspring	5	8.958		13.958	7.6	2.58	1.78
Athlete 101	female	Elite	18.55	221	Handspring	5	8.733		13.733	7.5	2.32	1.67
Athlete 115	female	Elite	17.20	221	Handspring	5	8.6		13.6	7.6	2.25	1.16
Athlete 120	female	Elite	21.19	221	Handspring	5	8.633		13.633	7.6	2.29	1.68
Athlete 57	female	Junior	14.77	221	Handspring	5	8.366	-0.1	13.266	7.9	2.37	1.75
Athlete 122	female	Elite	21.13	221	Handspring	5	8.766		13.766	7.7	2.48	1.89
Athlete 134	female	Elite	24.72	221	Handspring	5	8.966		13.966	7.8	2.36	2.14
Athlete 66	female	Junior	14.82	221	Handspring	5	8.9		13.9	7.9	2.52	1.56
Athlete 178	female	Elite	25.56	221	Handspring	5	8.633	-0.3	13.333	7.9	2.43	1.46
Athlete 110	female	Junior	14.95	221	Handspring	5	8.866		13.866	7.8	2.46	1.93
Athlete 155	female	Junior	14.60	221	Handspring	5	9		14	7.8	2.44	2.30
Athlete 156	female	Junior	13.61	221	Handspring	5	7.866	-0.1	12.766	7.5	2.36	1.79
Athlete 190	female	Junior	13.59	221	Handspring	5	9.033		14.033	7.8	2.57	1.48
Athete 212	male	junior	15.56	225	Tsukahara	3.6	8.866		12.466	7.5	2.40	1.99
Athlete 254	male	junior	14.60	225	Tsukahara	3.6	8.933		12.533	7.3	2.33	1.71
Athlete 286	male	junior	16.70	225	Tsukahara	3.6	8.466	-0.1	11.966	7.8	2.59	1.91
Athlete 305	male	junior	16.01	225	Tsukahara	3.6	8.533		12.133	8	2.38	1.86
Athlete 322	male	junior	14.73	225	Tsukahara	3.6	8.733		12.333	7	2.21	1.27
Athlete 351	male	junior	15.16	225	Tsukahara	3.6	8.433	-0.3	11.733	6.9	2.26	1.54
Athlete 360	male	junior	16.44	225	Tsukahara	3.6	8.766		12.366	7.4	2.51	1.86
Athlete 367	male	junior	15.25	225	Tsukahara	3.6	8.766	-0.1	12.266	7.7	2.57	2.16
Athlete 386	male	junior	16.37	225	Tsukahara	3.6	8.866		12.466	7.5	2.40	2.28
Athete 209	male	junior	18.20	226	Tsukahara	4	8.8		12.8	7.6	2.41	1.93
Athete 197	male	junior	18.00	227	Tsukahara	4.4	8.8		13.2	7.9	2.45	2.47
Athete 206	male	junior	16.32	227	Tsukahara	4.4	8.833		13.233	7.7	2.45	2.08
Athete 208	male	junior	16.42	227	Tsukahara	4.4	8.4		12.8	7.7	2.58	2.34
Athete 210	male	junior	18.41	227	Tsukahara	4.4	9.1		13.5	7.9	2.47	2.84
Athete 213	male	junior	17.04	227	Tsukahara	4.4	9		13.4	7.6	2.57	1.68
Athete 228	male	junior	15.50	227	Tsukahara	4.4	8.866		13.266	8.6	2.62	2.98
Athete 230	male	junior	16.84	227	Tsukahara	4.4	8.766		13.166	8.2	2.46	2.48
Athete 231	male	elite	23.97	227	Tsukahara	4.4	8	-0.1	12.3	7.5	2.67	1.65
Athete 232	male	junior	18.28	227	Tsukahara	4.4	8.866		13.266	7.8	2.56	1.44
Athete 235	male	elite	27.78	227	Tsukahara	4.4	9		13.4	7.8	2.42	2.57
Athlete 236	male	junior	17.06	227	Tsukahara	4.4	8.9		13.3	7.6	2.69	2.38
Athlete 238	male	junior	17.38	227	Tsukahara	4.4	8.6	-0.3	12.7	7.7	2.43	2.33
Athlete 239	male	junior	15.97	227	Tsukahara	4.4	8.966		13.366	7.8	2.53	2.01
Athlete 240	male	junior	17.15	227	Tsukahara	4.4	8.8		13.2	7.7	2.60	2.64
Athlete 246	male	junior	17.07	227	Tsukahara	4.4	8.9	-0.1	13.2	7.5	2.54	1.74
Athlete 247	male	junior	18.37	227	Tsukahara	4.4	8.733		13.133	7.8	2.52	2.15
Athlete 249	male	elite	20.25	227	Tsukahara	4.4	7.9	-0.3	12	7.9	2.46	2.42
Athlete 259	male	junior	16.92	227	Tsukahara	4.4	9.058		13.458	7.5	2.32	2.23
Athlete 267	male	junior	16.92	227	Tsukahara	4.4	8.833	-0.1	13.133	8.1	2.62	2.50
Athlete 268	male	junior	17.30	227	Tsukahara	4.4	8.933		13.333	7.8	2.51	2.72
Athlete 270	male	junior	16.88	227	Tsukahara	4.4	9.2		13.6	7.8	2.36	2.00
Athlete 274	male	junior	16.10	227	Tsukahara	4.4	9.033		13.433	8.1	2.59	3.00
Athlete 277	male	elite	19.90	227	Tsukahara	4.4	8.95		13.35	8.1	2.58	2.01
Athlete 281	male	junior	15.36	227	Tsukahara	4.4	8.866		13.266	8.1	2.56	2.37
Athlete 282	male	junior	16.05	227	Tsukahara	4.4	8.9		13.3	8.1	2.74	2.04
Athlete 285	male	junior	16.77	227	Tsukahara	4.4	9.033		13.433	7.7	2.48	2.23
Athlete 287	male	junior	16.53	227	Tsukahara	4.4	9.133		13.533	7.7	2.65	2.23
Athlete 294	male	junior	16.27	227	Tsukahara	4.4	8.933		13.333	8.1	2.72	2.56
Athlete 296	male	junior	16.82	227	Tsukahara	4.4	8.9		13.3	7.6	2.74	1.62
Athlete 300	male	junior	17.68	227	Tsukahara	4.4	8.866		13.266	8	2.73	2.91
Athlete 301	male	junior	17.65	227	Tsukahara	4.4	8.966		13.366	7.9	2.46	1.67
Athlete 303	male	junior	16.43	227	Tsukahara	4.4	8.833		13.233	8	2.49	2.12
Athlete 309	male	junior	16.52	227	Tsukahara	4.4	8.966	-0.1	13.266	8.1	2.47	2.74
Athlete 310	male	junior	17.92	227	Tsukahara	4.4	8.933		13.333	7.9	2.58	2.59
Athlete 314	male	junior	18.41	227	Tsukahara	4.4	9	-0.3	13.1	7.9		
Athlete 329	male	junior	14.53	227	Tsukahara	4.4	8.733		13.133	7.6	2.48	2.36
Athlete 331	male	junior	16.42	227	Tsukahara	4.4	9.1		13.5	7.7	2.56	2.14
Athlete 337	male	junior	16.97	227	Tsukahara	4.4	9.066		13.466	7.8	2.45	2.34
Athlete 339	male	junior	15.66	227	Tsukahara	4.4	8.933		13.333	7.8	2.76	2.45
Athlete 340	male	elite	21.83	227	Tsukahara	4.4	9.033	-0.1	13.333	8.2	2.52	3.01
Athlete 342	male	junior	16.34	227	Tsukahara	4.4	8.9		13.3	7.8	2.45	2.12
Athlete 343	male	elite	22.42	227	Tsukahara	4.4	8.666	-0.3	12.766	7.7	2.49	2.00
Athlete 345	male	junior	16.78	227	Tsukahara	4.4	9.3		13.7	8.2	2.72	3.20
Athlete 348	male	junior	15.54	227	Tsukahara	4.4	8.933		13.333	7.8	2.63	1.77
Athlete 356	male	junior	17.64	227	Tsukahara	4.4	9.1		13.5	7.7		
Athlete 361	male	elite	19.53	227	Tsukahara	4.4	8.916		13.316	7.9	2.47	2.07
Athlete 364	male	junior	17.57	227	Tsukahara	4.4	8.966		13.366	7.9		
Athlete 375	male	junior	17.36	227	Tsukahara	4.4	8.9		13.3	7.9	2.63	2.00
Athlete 377	male	junior	18.03	227	Tsukahara	4.4	9.066		13.466	8.4	2.74	2.76
Athlete 381	male	junior	14.29	227	Tsukahara	4.4	8.733		13.133	7.9	2.61	2.34
Athlete 387	male	junior	17.74	227	Tsukahara	4.4	8.9		13.3	7.6	2.42	2.63
Athlete 391	male	elite	22.04	227	Tsukahara	4.4	9.1		13.5	8	2.70	2.92
Athlete 401	male	junior	18.41	227	Tsukahara	4.4	9.1		13.5	8	2.50	2.63
Athlete 403	male	elite	19.74	227	Tsukahara	4.4	9		13.4	7.6	2.47	1.85

Athlete Nr	Gender	Category	Age	Vault Nr	Vault Group	D-Score	E-Score	Penalty	F-Score	v_{end}	h_{max}	L
Athlete 406	male	junior	18.13	227	Tsukahara	4.4	8.866		13.266	8.1	2.57	2.66
Athete 233	male	junior	18.31	228	Tsukahara	4.8	8.766	-0.3	13.266	7.8	2.55	1.94
Athlete 245	male	junior	15.79	228	Tsukahara	4.8	8.9		13.7	8.2	2.63	2.84
Athlete 265	male	junior	15.33	228	Tsukahara	4.8	8.666		13.466	8.2	2.56	2.69
Athlete 266	male	junior	17.38	228	Tsukahara	4.8	7.9	-0.1	12.6	8.4	2.63	2.78
Athlete 288	male	junior	15.15	228	Tsukahara	4.8	8.666		13.466	8	2.37	2.15
Athlete 291	male	junior	15.47	228	Tsukahara	4.8	8.5	-0.3	13	8.2	2.81	1.94
Athlete 323	male	junior	16.05	228	Tsukahara	4.8	7.933	-0.3	12.433	8.2	2.63	2.70
Athlete 336	male	junior	16.81	228	Tsukahara	4.8	9.1		13.9	8.6	2.67	3.04
Athlete 350	male	elite	22.05	228	Tsukahara	4.8	8.7	-0.3	13.2	8.1	2.53	2.56
Athlete 355	male	elite	20.80	228	Tsukahara	4.8	9		13.8	8.4	2.70	2.62
Athlete 378	male	junior	16.47	228	Tsukahara	4.8	7.8		12.6	7.8	2.45	2.64
Athete 199	male	junior	17.62	229	Tsukahara	5.2	8.8		14	8.1	2.67	2.75
Athete 202	male	elite	29.53	229	Tsukahara	5.2	8.833		14.033	8.1	2.65	2.34
Athete 203	male	elite	22.16	229	Tsukahara	5.2	8.066		13.266	8	2.59	1.92
Athete 207	male	junior	18.18	229	Tsukahara	5.2	7.466		12.666	8.4	2.64	3.36
Athete 218	male	elite	19.25	229	Tsukahara	5.2	8.666	-0.1	13.766	8.5	2.73	2.61
Athete 220	male	junior	17.68	229	Tsukahara	5.2	9.1		14.3	8.4	2.76	2.49
Athete 225	male	junior	17.29	229	Tsukahara	5.2	9.066		14.266	8	2.75	2.32
Athete 226	male	junior	17.60	229	Tsukahara	5.2	8.683	-0.3	13.583	8.1	2.68	2.59
Athlete 237	male	elite	21.50	229	Tsukahara	5.2	8.933		14.133	8.2	2.69	2.48
Athlete 243	male	elite	18.95	229	Tsukahara	5.2	8.866	-0.1	13.966	8	2.57	2.78
Athlete 248	male	elite	19.91	229	Tsukahara	5.2	8.8		14	8	2.68	2.23
Athlete 250	male	elite	22.39	229	Tsukahara	5.2	8.3	-0.3	13.2	8	2.65	2.59
Athlete 251	male	junior	17.48	229	Tsukahara	5.2	9		14.2	8	2.63	2.58
Athlete 255	male	junior	17.86	229	Tsukahara	5.2	8.966		14.166	8.1	2.67	2.14
Athlete 257	male	junior	17.84	229	Tsukahara	5.2	9.033		14.233	7.8	2.66	2.60
Athlete 258	male	elite	20.13	229	Tsukahara	5.2	8.866	-0.1	13.966	8.2	2.65	2.87
Athlete 262	male	junior	18.13	229	Tsukahara	5.2	9.1		14.3	7.9	2.59	2.21
Athlete 263	male	junior	17.89	229	Tsukahara	5.2	8.833		14.033	8	2.70	3.11
Athlete 271	male	elite	23.61	229	Tsukahara	5.2	9.033	-0.1	14.133	8	2.50	2.58
Athlete 272	male	elite	20.23	229	Tsukahara	5.2	8.633		13.833	7.8	2.53	2.36
Athlete 276	male	junior	15.80	229	Tsukahara	5.2	9.1		14.3	7.8	2.53	2.09
Athlete 289	male	elite	20.53	229	Tsukahara	5.2	9	-0.3	13.9	8.1	2.70	2.88
Athlete 292	male	elite	31.79	229	Tsukahara	5.2	9.3		14.5	8.6	2.79	2.42
Athlete 293	male	elite	18.70	229	Tsukahara	5.2	9		14.2	8		
Athlete 297	male	elite	20.26	229	Tsukahara	5.2	8.2	-0.1	13.3	8.1	2.64	2.88
Athlete 298	male	junior	18.37	229	Tsukahara	5.2	9.066		14.266	8.5	2.71	3.07
Athlete 299	male	junior	18.12	229	Tsukahara	5.2	8.933		14.133	7.6	2.55	2.38
Athlete 302	male	junior	18.40	229	Tsukahara	5.2	8.666	-0.3	13.566	7.7	2.68	2.06
Athlete 306	male	junior	16.84	229	Tsukahara	5.2	8.833		14.033	8.3	2.47	2.55
Athlete 307	male	elite	20.29	229	Tsukahara	5.2	8.933		14.133	8.2	2.73	2.66
Athlete 308	male	junior	17.31	229	Tsukahara	5.2	8.7	-0.1	13.8	8.1	2.69	2.99
Athlete 311	male	junior	15.70	229	Tsukahara	5.2	9.1		14.3	8.1	2.42	2.60
Athlete 313	male	elite	22.04	229	Tsukahara	5.2	9.033		14.233	7.9	2.62	2.05
Athlete 318	male	junior	17.49	229	Tsukahara	5.2	9.066		14.266	8.2	2.60	2.91
Athlete 320	male	junior	16.86	229	Tsukahara	5.2	8.766		13.966	7.7	2.53	2.11
Athlete 324	male	junior	16.21	229	Tsukahara	5.2	8.7		13.9	7.8	2.62	1.99
Athlete 327	male	junior	14.88	229	Tsukahara	5.2	9.066		14.266	7.7	2.31	2.18
Athlete 328	male	elite	18.84	229	Tsukahara	5.2	9.133		14.333	8.4	2.68	3.26
Athlete 333	male	elite	21.80	229	Tsukahara	5.2	9.066		14.266	8.4	2.88	3.02
Athlete 334	male	elite	24.51	229	Tsukahara	5.2	9	-0.1	14.1	8.4	2.61	2.58
Athlete 338	male	elite	30.64	229	Tsukahara	5.2	8	-0.3	12.9	7.8	2.46	2.14
Athlete 341	male	junior	17.09	229	Tsukahara	5.2	8.9		14.1	8.3	2.80	2.07
Athlete 354	male	junior	16.86	229	Tsukahara	5.2	8.9		14.1	8.3	2.71	2.96
Athlete 359	male	elite	19.15	229	Tsukahara	5.2	8.8	-0.1	13.9	8.5	2.92	2.67
Athlete 362	male	elite	25.36	229	Tsukahara	5.2	8.8		14	8.2	2.66	2.36
Athlete 365	male	junior	17.08	229	Tsukahara	5.2	8.966		14.166	8.1	2.58	2.52
Athlete 366	male	junior	17.24	229	Tsukahara	5.2	8.9		14.1	8.5	2.86	2.59
Athlete 368	male	junior	17.92	229	Tsukahara	5.2	8.9		14.1	8	2.45	2.03
Athlete 371	male	elite	22.03	229	Tsukahara	5.2	8.7		13.9	8.3	2.62	2.78
Athlete 372	male	elite	19.17	229	Tsukahara	5.2	9.1		14.3	8.4	2.68	2.75
Athlete 374	male	elite	18.71	229	Tsukahara	5.2	8.766		13.966	8.2	2.68	3.10
Athlete 383	male	junior	18.21	229	Tsukahara	5.2	7.666	-0.3	12.566	8	2.66	2.83
Athlete 389	male	elite	21.65	229	Tsukahara	5.2	9		14.2	8.3	2.69	3.11
Athlete 390	male	junior	16.45	229	Tsukahara	5.2	8.733		13.933	7.8	2.78	2.34
Athlete 393	male	junior	18.19	229	Tsukahara	5.2	9.1	-0.1	14.2	8.2	2.53	2.39
Athlete 394	male	junior	17.28	229	Tsukahara	5.2	8.8	-0.1	13.9	7.5	2.75	1.78
Athlete 395	male	junior	16.94	229	Tsukahara	5.2	8.9	-0.3	13.8	8.1	2.62	2.66
Athlete 397	male	elite	22.76	229	Tsukahara	5.2	9		14.2	7.9	2.63	2.00
Athete 196	male	elite	23.17	230	Tsukahara	5.6	9.266		14.866	8.1	2.71	2.38
Athete 207	male	elite	22.84	230	Tsukahara	5.6	9.066		14.666	8.9	2.66	2.65
Athete 216	male	elite	28.14	230	Tsukahara	5.6	9.066		14.666	8.1	2.68	2.27
Athete 221	male	junior	17.98	230	Tsukahara	5.6	9.166		14.766	8.4	2.82	2.07
Athete 222	male	elite	22.68	230	Tsukahara	5.6	8.933		14.533	8.5	2.68	2.71
Athete 223	male	elite	23.51	230	Tsukahara	5.6	9.266	-0.1	14.766	8.6	2.80	2.92
Athete 229	male	elite	25.80	230	Tsukahara	5.6	9.066		14.666	8.3	2.62	2.90
Athlete 260	male	elite	20.53	230	Tsukahara	5.6	9.333		14.933	8.6	2.75	2.56
Athlete 280	male	junior	18.23	230	Tsukahara	5.6	9.033		14.633	8.1	2.69	1.75
Athlete 315	male	junior	17.96	230	Tsukahara	5.6	9.033		14.633	8.4	2.73	2.67
Athlete 319	male	junior	16.84	230	Tsukahara	5.6	7.866		13.466	8	2.63	2.04
Athlete 325	male	elite	23.33	230	Tsukahara	5.6	9.166		14.766	8.3	2.66	2.91
Athlete 330	male	elite	19.88	230	Tsukahara	5.6	9.1		14.7	8.2	2.85	2.82
Athlete 353	male	junior	18.39	230	Tsukahara	5.6	8.766	-0.1	14.266	8.1	2.54	2.35
Athlete 369	male	elite	24.23	230	Tsukahara	5.6	8.8	-0.1	14.3	8.4	2.78	2.53
Athlete 379	male	elite	22.62	230	Tsukahara	5.6	9.2		14.8	8.2	2.69	2.20
Athlete 382	male	elite	25.65	230	Tsukahara	5.6	8.866	-0.1	14.366	8	2.81	2.64
Athlete 388	male	elite	26.51	230	Tsukahara	5.6	9.166	-0.1	14.666	8.3	2.80	2.44
Athlete 399	male	elite	24.02	230	Tsukahara	5.6	8.166	-0.3	13.466	8.6	2.71	3.23
Athlete 400	male	elite	20.37	230	Tsukahara	5.6	9.2		14.8		2.68	3.10
Athete 234	male	elite	23.81	231	Tsukahara	6	9.166	-0.1	15.066	9	3.01	2.21
Athlete 326	male	elite	19.29	231	Tsukahara	6	9.166		15.166	8.2	2.90	2.30

Athlete Nr	Gender	Category	Age	Vault Nr	Vault Group	D-Score	E-Score	Penalty	F-Score	v_{end}	h_{max}	L
Athlete 352	male	junior	17.82	231	Tsukahara	6	9.366		15.366	8.1	2.82	2.38
Athlete 398	male	elite	22.67	231	Tsukahara	6	8.6	-0.1	14.5	8.5	2.79	3.09
Athlete 402	male	elite	18.56	231	Tsukahara	6	8.933		14.933	8.8	2.72	2.38
Athlete 28	female	Elite	15.75	231	Handspring	5.4	8.933		14.333	7.9	2.70	1.55
Athlete 78	female	Elite	24.30	231	Handspring	5.4	9		14.4	7.8	2.48	1.71
Athlete 173	female	Elite	27.68	231	Handspring	5.4	8.766		14.166	8	2.40	1.72
Athlete 10	female	Elite	22.13	232	Handspring	5.8	8.966		14.766	8.3	2.49	2.03
Athlete 162	female	Elite	22.21	233	Handspring	6.2	9.466		15.666	8.2	2.83	2.00
Athlete 279	male	junior	18.17	237	Tsukahara	5.6	8.866		14.466	8.4	2.81	3.35
Athete 198	male	elite	23.82	238	Tsukahara	6.4	9.333		15.733	8.5	2.91	2.88
Athete 217	male	elite	24.27	243	Tsukahara	6	8.033	-0.1	13.933	7.8	2.66	2.63
Athlete 275	male	elite	19.36	243	Tsukahara	6	9.1		15.1	8.4	2.91	2.69
Athlete 321	male	elite	26.15	243	Tsukahara	6	9.066		15.066	8.6	2.92	2.85
Athlete 349	male	elite	23.61	243	Tsukahara	6	8.1	-0.1	14	8.3	2.85	3.16
Athlete 407	male	elite	21.66	243	Tsukahara	6	9.1	-0.1	15	8.4	2.79	2.37
Athlete 7	female	Elite	16.48	310	Tsukahara	4	8.166		12.166	7.1	2.19	1.29
Athlete 189	female	Elite	17.55	310	Tsukahara	4	7.833		11.833	6.8	1.99	1.72
Athlete 131	female	Junior	13.63	310	Tsukahara	4	8.266		12.266	6.5	2.18	1.69
Athete 227	male	junior	13.73	311	Yurchenko	4.4	8.8	-0.3	12.9	7.2	2.41	1.56
Athlete 146	female	Junior	14.17	312	Tsukahara	4.6	8.4		13	7.4	2.35	1.95
Athlete 184	female	Junior	14.78	312	Tsukahara	4.6	8.633		13.233	7.5	2.28	1.46
Athlete 380	male	elite	27.29	317	Yurchenko	6	9.1	-0.1	15	7.8	2.98	2.56
Athlete 22	female	Elite	20.20	320	Tsukahara	4.2	8.4		12.6	7.5	2.22	2.09
Athlete 14	female	Elite	18.16	320	Tsukahara	4.2	8.5		12.7	7.8	2.18	1.57
Athlete 16	female	Junior	14.79	320	Tsukahara	4.2	7.666		11.866	7.7	2.34	1.86
Athlete 17	female	Elite	17.16	320	Tsukahara	4.2	7.333		11.533		2.22	1.46
Athlete 35	female	Elite	17.41	320	Tsukahara	4.2	8.333		12.533	7	2.35	1.45
Athlete 42	female	Elite	17.21	320	Tsukahara	4.2	8.233		12.433	6.9	2.25	1.74
Athlete 59	female	Elite	16.37	320	Tsukahara	4.2	7.933		12.133	7	2.13	1.53
Athlete 61	female	Elite	18.18	320	Tsukahara	4.2	7.4		11.6	7.3	2.50	1.93
Athlete 67	female	Elite	16.08	320	Tsukahara	4.2	8.6		12.8	7.6	2.39	1.67
Athlete 86	female	Elite	16.98	320	Tsukahara	4.2	7.5		11.7		2.28	1.66
Athlete 46	female	Junior	13.74	320	Tsukahara	4.2	8.566		12.766	7.2	2.34	1.42
Athlete 112	female	Elite	17.40	320	Tsukahara	4.2	8.333		12.533	7	2.25	1.91
Athlete 63	female	Junior	13.98	320	Tsukahara	4.2	8.666		12.866	7.4	2.37	2.12
Athlete 138	female	Elite	16.96	320	Tsukahara	4.2	8.5		12.7	7.1	2.17	2.08
Athlete 148	female	Elite	16.85	320	Tsukahara	4.2	8.7		12.9	7.3	2.27	1.65
Athlete 168	female	Elite	16.37	320	Tsukahara	4.2	8.466		12.666	6.8	2.18	1.47
Athlete 90	female	Junior	14.92	320	Tsukahara	4.2	8.366		12.566	7.3	2.17	2.08
Athlete 6	female	Junior	15.08	320	Tsukahara	4.2	8.166		12.366	7	2.18	1.16
Athlete 12	female	Junior	13.61	320	Tsukahara	4.2	8.7		12.9	7.3	2.36	1.95
Athlete 21	female	Junior	14.72	320	Tsukahara	4.2	8.633		12.833	7.4	2.31	1.84
Athlete 40	female	Junior	14.42	320	Tsukahara	4.2	8.7		12.9	7.2	2.37	2.00
Athlete 43	female	Junior	14.32	320	Tsukahara	4.2	8.466		12.666	7.4	2.21	1.80
Athlete 51	female	Junior	14.14	320	Tsukahara	4.2	8.366	-0.1	12.466	7.3	2.26	1.52
Athlete 52	female	Junior	14.04	320	Tsukahara	4.2	8.566		12.766	7	2.26	1.83
Athlete 77	female	Junior	14.62	320	Tsukahara	4.2	8.266		12.466	7.3	2.33	2.17
Athlete 131	female	Junior	13.63	320	Tsukahara	4.2	7.633		11.833	6.6	2.24	1.56
Athlete 81	female	Junior	13.84	320	Tsukahara	4.2	8.333	-0.1	12.433	6.7	2.21	1.16
Athlete 87	female	Junior	13.70	320	Tsukahara	4.2	8.533		12.733	7.6	2.32	1.69
Athlete 88	female	Junior	15.38	320	Tsukahara	4.2	8.533		12.733	6.7	2.30	1.57
Athlete 89	female	Junior	15.30	320	Tsukahara	4.2	8.466		12.666	7.4	2.31	2.00
Athlete 144	female	Junior	13.70	320	Tsukahara	4.2	8.6		12.8	7.3	2.34	1.43
Athlete 108	female	Junior	15.20	320	Tsukahara	4.2	8.5		12.7	7	2.26	1.97
Athlete 113	female	Junior	15.01	320	Tsukahara	4.2	8.5		12.7	7.1	2.20	1.37
Athlete 141	female	Junior	14.79	320	Tsukahara	4.2	8.033		12.233	7.5	2.39	2.31
Athlete 145	female	Junior	13.93	320	Tsukahara	4.2	8.633		12.833	7.4	2.23	1.91
Athlete 164	female	Junior	14.37	320	Tsukahara	4.2	8.233	-0.1	12.333	7	2.23	1.87
Athlete 169	female	Junior	14.69	320	Tsukahara	4.2	8.633		12.833	7	2.31	1.52
Athlete 172	female	Junior	13.70	320	Tsukahara	4.2	8.7		12.9	6.9	2.22	1.64
Athlete 175	female	Junior	13.62	320	Tsukahara	4.2	8.7		12.9	7.3	2.32	1.60
Athlete 179	female	Junior	13.51	320	Tsukahara	4.2	8.366		12.566	7.1	2.22	2.00
Athlete 192	female	Junior	15.31	320	Tsukahara	4.2	8.666		12.866	7.5	2.42	2.16
Athlete 195	female	Junior	13.72	320	Tsukahara	4.2	8.5		12.7	7	2.33	1.77
Athete 219	male	junior	17.22	321	Yurchenko	4.4	8.9		13.3	7.2	2.46	2.38
Athlete 241	male	junior	15.52	321	Yurchenko	4.4	8.8		13.2	7.3	2.49	1.31
Athlete 333	male	elite	21.80	321	Yurchenko	4.4	9		13.4	7.1	2.48	2.23
Athlete 363	male	junior	17.58	321	Yurchenko	4.4	8.866		13.266	6.9	2.66	1.79
Athete 201	male	elite	20.78	322	Yurchenko	4.8	9.3		14.1		2.60	2.27
Athlete 308	male	junior	17.31	322	Yurchenko	4.8	9		13.8	7.6	2.61	1.93
Athete 215	male	elite	30.27	323	Yurchenko	5.2	9.133		14.333	7.4	2.61	2.06
Athete 224	male	junior	18.31	323	Yurchenko	5.2	7.8		13	7.1	2.57	1.78
Athete 233	male	junior	18.31	323	Yurchenko	5.2	8.9		14.1	7	2.48	1.66
Athlete 242	male	elite	19.57	323	Yurchenko	5.2	9		14.2	6.9	2.77	1.55
Athlete 252	male	elite	24.57	323	Yurchenko	5.2	8.533	-0.1	13.633		2.71	1.72
Athlete 273	male	junior	17.88	323	Yurchenko	5.2	8.866	-0.3	13.766	7.6	2.64	2.30
Athlete 278	male	elite	22.44	323	Yurchenko	5.2	9.183		14.383	7.5	2.78	2.08
Athlete 284	male	elite	19.48	323	Yurchenko	5.2	8.8	-0.1	13.9	7.2	2.72	2.22
Athlete 288	male	junior	15.15	323	Yurchenko	5.2	8.733		13.933	7.7	2.41	2.01
Athlete 295	male	elite	20.45	323	Yurchenko	5.2		9.166	14.366	7.6	2.72	2.11
Athlete 304	male	elite	25.95	323	Yurchenko	5.2	9.1		14.3	7.2	2.74	1.70
Athlete 316	male	elite	33.18	323	Yurchenko	5.2	9.366		14.566	7.2	2.76	1.95
Athlete 317	male	elite	28.97	323	Yurchenko	5.2	9.033		14.233	7.1	2.65	2.08
Athlete 319	male	junior	16.84	323	Yurchenko	5.2	8.966	-0.1	14.066	6.7	2.70	1.43
Athlete 323	male	junior	16.05	323	Yurchenko	5.2	8.933		14.133	7.7	2.59	2.76
Athlete 332	male	junior	16.35	323	Yurchenko	5.2	9.166		14.366	7.3	2.78	1.54
Athlete 357	male	junior	16.20	323	Yurchenko	5.2	9.066	-0.1	14.166	7.7	2.71	2.41
Athlete 392	male	elite	19.13	323	Yurchenko	5.2	9.2		14.4	7.7	2.77	2.52
Athlete 404	male	elite	20.91	323	Yurchenko	5.2	9.133		14.333	6.9	2.56	2.13
Athete 204	male	elite	22.16	325	Yurchenko	5.6	9.033	-0.1	14.533	7.6	2.89	2.01
Athlete 347	male	elite	25.55	325	Yurchenko	5.6	9.1		14.7	7.3	2.82	1.50
Athlete 396	male	elite	20.37	325	Yurchenko	5.6	8.5	-0.3	13.8	8	2.80	2.27

Athlete Nr	Gender	Category	Age	Vault Nr	Vault Group	D-Score	E-Score	Penalty	F-Score	v_{end}	h_{max}	L
Athlete 31	female	Elite	17.13	330	Tsukahara	4.6	8.5		13.1	7.3	2.14	1.66
Athlete 47	female	Elite	23.72	330	Tsukahara	4.6	8.883		13.483	7.5	2.38	1.66
Athlete 84	female	Elite	16.16	330	Tsukahara	4.6	9.033		13.633	7.7	2.47	2.12
Athlete 75	female	Elite	23.24	330	Tsukahara	4.6	9.058		13.658	8.1	2.60	2.14
Athlete 30	female	Junior	13.80	330	Tsukahara	4.6	8.833		13.433	7.5	2.39	1.96
Athlete 124	female	Elite	17.29	330	Tsukahara	4.6	8.6		13.2	7.4	2.24	1.71
Athlete 73	female	Junior	14.44	330	Tsukahara	4.6	8.633		13.233	7.4	2.38	2.01
Athlete 187	female	Elite	15.51	330	Tsukahara	4.6	8.466		13.066	7.2	2.21	1.74
Athlete 62	female	Elite	20.12	332	Tsukahara	5.2	8.466		13.666	7.9	2.40	2.15
Athlete 171	female	Elite	16.77	332	Tsukahara	5.2	8.658		13.858	7.7	2.30	1.99
Athlete 78	female	Elite	24.30	332	Tsukahara	5.2	9.033		14.233	7.8	2.30	2.17
Athlete 122	female	Elite	21.13	332	Tsukahara	5.2	8.8		14	7.7	2.28	1.92
Athlete 74	female	Junior	13.64	332	Tsukahara	5.2	8.7		13.9		2.20	1.80
Athlete 134	female	Elite	24.72	332	Tsukahara	5.2	8.633		13.833	7.6	2.34	2.50
Athlete 173	female	Elite	27.68	332	Tsukahara	5.2	8.766	-0.1	13.866	7.9	2.45	2.01
Athlete 157	female	Junior	15.28	332	Tsukahara	5.2	8.733		13.933	8.1	2.35	2.31
Athlete 168	female	Elite	16.37	410	Yurchenko	3.8	8.433		12.233	6.6	2.32	1.68
Athlete 149	female	Junior	14.94	412	Yurchenko	4.4	8.566		12.966	7.3	2.34	1.91
Athlete 85	female	Elite	15.70	420	Yurchenko	4	8.633		12.633	6.9	2.61	1.82
Athlete 3	female	Junior	14.37	420	Yurchenko	4	8.6	-0.3	12.3	7	2.43	1.37
Athlete 80	female	Junior	14.55	420	Yurchenko	4	8.3		12.3	6.8	2.38	1.33
Athlete 97	female	Junior	13.71	420	Yurchenko	4	8.6		12.6	7.2	2.49	2.00
Athlete 139	female	Junior	13.97	420	Yurchenko	4	7.733		11.733	6.9	2.40	1.18
Athlete 147	female	Junior	14.27	420	Yurchenko	4	8.566		12.566	7.2	2.32	1.75
Athlete 183	female	Junior	13.94	420	Yurchenko	4	8.7		12.7	6.8	2.37	1.54
Athete 198	male	elite	23.82	425	Yurchenko	6.2	9.1	-0.1	15.2	8	2.67	2.84
Athlete 244	male	elite	35.46	425	Yurchenko	6.2	8.833	-0.1	14.933	7.5	2.64	2.66
Athlete 11	female	Elite	16.31	430	Yurchenko	4.4	8.633		13.033	7.5	2.27	2.29
Athlete 32	female	Elite	17.32	430	Yurchenko	4.4	8.6		13	7.4	2.30	1.43
Athlete 123	female	Elite	15.93	430	Yurchenko	4.4	8.6		13	7.4	2.26	2.04
Athlete 140	female	Elite	23.50	430	Yurchenko	4.4	8.7		13.1		2.24	1.61
Athlete 158	female	Elite	16.92	430	Yurchenko	4.4	8.933		13.333	7	2.51	1.67
Athlete 165	female	Elite	23.66	430	Yurchenko	4.4	8.7		13.1	7.2	2.45	1.82
Athlete 4	female	Junior	14.89	430	Yurchenko	4.4	7.966	-0.1	12.266	7.1	2.44	1.42
Athlete 5	female	Junior	15.10	430	Yurchenko	4.4	8.5		12.9	7.5	2.50	1.60
Athlete 19	female	Junior	14.94	430	Yurchenko	4.4	8.7		13.1	6.9	2.33	1.92
Athlete 23	female	Junior	14.41	430	Yurchenko	4.4	8.3		12.7	7.1	2.47	1.38
Athlete 45	female	Junior	15.05	430	Yurchenko	4.4	8.7		13.1	7.1	2.49	1.61
Athlete 55	female	Junior	14.85	430	Yurchenko	4.4	8.633		13.033	7.2	2.42	1.33
Athlete 100	female	Junior	14.19	430	Yurchenko	4.4	8.466		12.866	7	2.34	1.74
Athlete 109	female	Junior	15.20	430	Yurchenko	4.4	8.766		13.166	7.1	2.38	1.95
Athlete 148	female	Elite	16.85	430	Yurchenko	4.4	8.7		13.1	7.2	2.41	1.59
Athlete 126	female	Junior	14.81	430	Yurchenko	4.4	8.9		13.3	6.9	2.45	1.97
Athlete 128	female	Junior	15.38	430	Yurchenko	4.4	8.6		13	6.9	2.32	1.95
Athlete 133	female	Junior	14.58	430	Yurchenko	4.4	8.733		13.133	7.3	2.49	1.85
Athlete 159	female	Elite	20.65	430	Yurchenko	4.4	8.766		13.166	6.9	2.34	1.80
Athlete 150	female	Junior	15.41	430	Yurchenko	4.4	8.566		12.966	6.8	2.19	1.75
Athlete 151	female	Junior	14.27	430	Yurchenko	4.4	8.766	-0.3	12.866	7.1	2.42	1.96
Athlete 154	female	Junior	15.18	430	Yurchenko	4.4	8.9		13.3	7.2	2.49	1.89
Athlete 160	female	Junior	15.36	430	Yurchenko	4.4	8.633		13.033	7.1	2.39	1.71
Athlete 194	female	Junior	13.62	430	Yurchenko	4.4	8.633		13.033	6.6	2.44	1.86
Athlete 2	female	Elite	18.02	432	Yurchenko	5	8.933		13.933	7.1	2.51	1.59
Athlete 8	female	Elite	15.53	432	Yurchenko	5	8.6		13.6	7.2	2.42	1.81
Athlete 15	female	Elite	17.29	432	Yurchenko	5	8.9	-0.3	13.6	7.5	2.68	1.31
Athlete 25	female	Elite	21.54	432	Yurchenko	5	9.241		14.241	7.6	2.63	1.94
Athlete 26	female	Elite	16.35	432	Yurchenko	5	8.966		13.966	7.2	2.43	1.66
Athlete 27	female	Elite	27.38	432	Yurchenko	5	8.966		13.966	7.2	2.42	1.87
Athlete 18	female	Elite	24.31	432	Yurchenko	5	9.033		14.033	7.2	2.53	1.79
Athlete 37	female	Elite	17.69	432	Yurchenko	5	9		14	7.3	2.55	1.65
Athlete 41	female	Elite	17.31	432	Yurchenko	5	8.9		13.9	7.5	2.72	1.72
Athlete 49	female	Elite	18.34	432	Yurchenko	5	8.866		13.866	7	2.59	1.83
Athlete 50	female	Elite	16.63	432	Yurchenko	5	9.166		14.166	7.2	2.48	1.48
Athlete 72	female	Elite	21.15	432	Yurchenko	5	8.6		13.6	7.6	2.33	2.15
Athlete 79	female	Elite	18.49	432	Yurchenko	5	8.966		13.966	7.2	2.48	1.71
Athlete 91	female	Elite	25.31	432	Yurchenko	5	8.9		13.9	7.4	2.51	1.65
Athlete 93	female	Elite	15.76	432	Yurchenko	5	8.7		13.7	7.5	2.30	2.19
Athlete 47	female	Elite	23.72	432	Yurchenko	5	8.966		13.966	7.1	2.60	2.07
Athlete 104	female	Elite	20.41	432	Yurchenko	5	9.166		14.166	7.4	2.72	2.38
Athlete 111	female	Elite	15.80	432	Yurchenko	5	8.9		13.9	7.6	2.49	1.78
Athlete 114	female	Elite	19.24	432	Yurchenko	5	8.8	-0.1	13.7	7.3	2.52	1.97
Athlete 121	female	Elite	24.56	432	Yurchenko	5	8.933		13.933	7.4	2.41	1.66
Athlete 58	female	Elite	17.98	432	Yurchenko	5	8.533	-0.3	13.233	7	2.34	1.55
Athlete 130	female	Elite	18.48	432	Yurchenko	5	9.2		14.2	7.1	2.48	1.94
Athlete 132	female	Elite	15.71	432	Yurchenko	5	9.166		14.166	7.5	2.67	1.66
Athlete 153	female	Elite	24.76	432	Yurchenko	5	8.9		13.9	7.4	2.52	1.76
Athlete 74	female	Junior	13.64	432	Yurchenko	0	0		0	7.3	2.35	1.95
Athlete 180	female	Elite	17.25	432	Yurchenko	5	8.733		13.733	7.3	2.39	1.72
Athlete 185	female	Elite	16.61	432	Yurchenko	5	8.8		13.8	7.5	2.64	1.78
Athlete 193	female	Elite	17.47	432	Yurchenko	5	8.6		13.6	7	2.32	2.26
Athlete 92	female	Elite	23.11	432	Yurchenko	5	9.2		14.2	6.8	2.50	1.80
Athlete 9	female	Junior	13.64	432	Yurchenko	5	9.1		14.1	7.2	2.49	1.86
Athlete 98	female	Elite	23.45	432	Yurchenko	5	8.933		13.933	7.4	2.47	2.40
Athlete 24	female	Junior	14.95	432	Yurchenko	5	8.766		13.766	7.4	2.56	1.60
Athlete 33	female	Junior	14.50	432	Yurchenko	5	8.866		13.866	7.2	2.42	1.86
Athlete 34	female	Junior	15.42	432	Yurchenko	5	9.066		14.066	7.6	2.54	2.33
Athlete 36	female	Junior	14.32	432	Yurchenko	5	8.933		13.933	7.1	2.38	1.69
Athlete 38	female	Junior	14.64	432	Yurchenko	5	8.833		13.833	7.3	2.49	1.41
Athlete 54	female	Junior	14.53	432	Yurchenko	5	7.9		12.9	7.5	2.42	2.48
Athlete 56	female	Junior	15.07	432	Yurchenko	5	8.916		13.916	7.1	2.29	1.79
Athlete 60	female	Junior	14.24	432	Yurchenko	5	9		14	7.4	2.48	1.54
Athlete 69	female	Junior	14.86	432	Yurchenko	5	8.8		13.8	7.1	2.47	1.83
Athlete 70	female	Junior	14.93	432	Yurchenko	5	8.733		13.733	7.2	2.53	2.15

Athlete Nr	Gender	Category	Age	Vault Nr	Vault Group	D-Score	E-Score	Penalty	F-Score	v_{end}	h_{max}	L
Athlete 71	female	Junior	15.39	432	Yurchenko	5	9.033		14.033	7	2.55	1.85
Athlete 82	female	Junior	15.23	432	Yurchenko	5	8.966		13.966	7.3	2.42	1.98
Athlete 99	female	Junior	14.86	432	Yurchenko	5	9.066		14.066	7.4	2.56	2.04
Athlete 105	female	Junior	13.57	432	Yurchenko	5	8.866		13.866	7.6	2.43	2.10
Athlete 145	female	Junior	13.93	432	Yurchenko	5	8.633		13.633	7	2.39	1.80
Athlete 146	female	Junior	14.17	432	Yurchenko	5	8.566		13.566	7	2.33	1.97
Athlete 118	female	Junior	15.19	432	Yurchenko	5	9.1		14.1	7.6	2.56	1.80
Athlete 151	female	Junior	14.27	432	Yurchenko	5	8.8		13.8	7	2.41	1.64
Athlete 127	female	Junior	13.47	432	Yurchenko	5	8.9		13.9	7.2	2.47	1.67
Athlete 129	female	Junior	15.09	432	Yurchenko	0	0		0	7.2		
Athlete 156	female	Junior	13.61	432	Yurchenko	5	8.933		13.933	7.2	2.35	1.68
Athlete 135	female	Junior	14.75	432	Yurchenko	5	9.083		14.083	7.4	2.57	1.99
Athlete 137	female	Junior	14.84	432	Yurchenko	5	8.8		13.8	7.4	2.47	2.19
Athlete 152	female	Junior	15.42	432	Yurchenko	5	8.666	-0.1	13.566	7.4	2.54	1.66
Athlete 161	female	Junior	14.71	432	Yurchenko	5	8.933		13.933	7	2.44	2.38
Athlete 163	female	Junior	15.18	432	Yurchenko	5	8.8		13.8	7.1	2.37	1.85
Athlete 178	female	Elite	25.56	432	Yurchenko	5	8.833		13.833	7.4	2.69	1.67
Athlete 170	female	Junior	15.30	432	Yurchenko	5	8.658		13.658	6.9	2.54	1.56
Athlete 182	female	Junior	14.35	432	Yurchenko	5	8.566		13.566	7.2	2.27	1.26
Athlete 181	female	Junior	15.21	432	Yurchenko	5	8.9		13.9	7.1	2.53	1.98
Athlete 189	female	Elite	17.55	432	Yurchenko	5	8.7		13.7	7.1	2.41	1.67
Athlete 9	female	Junior	13.64	433	Yurchenko	5.3	8.933		14.233	7.2	2.57	1.61
Athlete 10	female	Elite	22.13	433	Yurchenko	5.3	8.933		14.233	7.3	2.55	1.98
Athlete 36	female	Junior	14.32	433	Yurchenko	5.3	8.8		14.1	7.3	2.31	2.02
Athlete 107	female	Elite	18.87	433	Yurchenko	5.3	8.933		14.233	7.3	2.42	1.47
Athlete 69	female	Junior	14.86	433	Yurchenko	5.3	8.733		14.033	7.2	2.52	2.00
Athlete 48	female	Junior	14.85	433	Yurchenko	5.3	9.066		14.366	7.2	2.53	1.52
Athlete 127	female	Junior	13.47	433	Yurchenko	5.3	9		14.3	7.3	2.47	1.58
Athlete 129	female	Junior	15.09	433	Yurchenko	5.3	7.866	-0.3	12.866		2.51	1.80
Athlete 96	female	Junior	14.90	433	Yurchenko	5.3	8.408	-0.1	13.608	7.3	2.20	2.21
Athlete 157	female	Junior	15.28	433	Yurchenko	5.3	8.7		14	7.4	2.56	1.98
Athlete 1	female	Elite	24.73	434	Yurchenko	5.8	9.166		14.966	7.4	2.64	1.78
Athlete 20	female	Elite	16.04	434	Yurchenko	5.8	8.9		14.7	7.3	2.69	1.67
Athlete 44	female	Elite	16.88	434	Yurchenko	5.8	9.233		15.033	7.9	2.64	1.97
Athlete 53	female	Elite	18.62	434	Yurchenko	5.8	8.933		14.733	7	2.40	1.51
Athlete 28	female	Elite	15.75	434	Yurchenko	5.8	9		14.8	7.7	2.88	1.23
Athlete 65	female	Elite	20.01	434	Yurchenko	5.8	9		14.8	7.6	2.59	1.84
Athlete 34	female	Junior	15.42	434	Yurchenko	5.8	8.766		14.566	7.5	2.44	2.91
Athlete 48	female	Junior	14.85	434	Yurchenko	5.8	8.9		14.7	7.3	2.56	1.32
Athlete 106	female	Elite	15.88	434	Yurchenko	5.8	9.166		14.966	7.4	2.57	2.25
Athlete 60	female	Junior	14.24	434	Yurchenko	5.8	9.133		14.933	7.5	2.53	1.77
Athlete 125	female	Elite	18.49	434	Yurchenko	5.8	8.866		14.666	7.4	2.55	2.11
Athlete 136	female	Elite	28.80	434	Yurchenko	5.8	9.341		15.141	7.4	2.76	2.03
Athlete 142	female	Elite	18.20	434	Yurchenko	5.8	8.566		14.366	7.5	2.47	1.82
Athlete 177	female	Elite	16.89	434	Yurchenko	5.8	8.966		14.766	7.4	2.46	1.84
Athlete 84	female	Elite	16.16	434	Yurchenko	5.8	9.333		15.133	7.5	2.65	2.06
Athlete 186	female	Elite	16.62	434	Yurchenko	5.8	8.733	-0.1	14.433	7.8	2.66	1.87
Athlete 99	female	Junior	14.86	434	Yurchenko	5.8	9.033		14.833	7.4	2.60	1.97
Athlete 162	female	Elite	22.21	434	Yurchenko	5.8	9.4		15.2	7.1	2.71	2.28
Athlete 114	female	Elite	19.24	510	Yurchenko	4.6	7.4	-0.3	11.7	7.2	2.38	1.72
Athlete 8	female	Elite	15.53	520	Yurchenko	4.8	7.4		12.2	7.2	2.34	2.05
Athlete 1	female	Elite	24.73	531	Yurchenko	5.6	8.941		14.541	7.3	2.27	1.99
Athlete 44	female	Elite	16.88	531	Yurchenko	5.6	9.1		14.7	8	2.50	2.32
Athlete 53	female	Elite	18.62	531	Yurchenko	5.6	8.766		14.366	7.1	2.26	1.55
Athlete 177	female	Elite	16.89	531	Yurchenko	5.6	8.5	-0.3	13.8	7.5	2.28	1.98
